# Adsorption Performance of Fe–Mn Polymer Nanocomposites for Arsenic Removal: Insights from Kinetic and Isotherm Models

**DOI:** 10.3390/ma17205089

**Published:** 2024-10-18

**Authors:** Jasmina Nikić, Malcolm Watson, Jovana Jokić Govedarica, Maja Vujić, Jovana Pešić, Srđan Rončević, Jasmina Agbaba

**Affiliations:** Department of Chemistry, Biochemistry and Environmental Protection, Faculty of Sciences, University of Novi Sad, Trg Dositeja Obradovića 3, 21000 Novi Sad, Serbia; jasmina.nikic@dh.uns.ac.rs (J.N.); jovanaj@dh.uns.ac.rs (J.J.G.); maja.loncarski@dh.uns.ac.rs (M.V.); jovana.pesic@dh.uns.ac.rs (J.P.); srdjan.roncevic@dh.uns.ac.rs (S.R.); jasmina.agbaba@dh.uns.ac.rs (J.A.)

**Keywords:** arsenic, drinking water, FMBO, nanocomposite, polymers, modelling

## Abstract

Global concern over arsenic contamination in drinking water necessitates innovative and sustainable remediation technologies. This study evaluates the adsorption performance of Fe–Mn binary oxide (FMBO) nanocomposites developed by coating polyethylene (PE) and polyethylene terephthalate (PET) with FMBO for the removal of As(III) and As(V) from water. Adsorption kinetics were rapid, with equilibrium achieved within 1–4 h depending on the material and pH. PET-FMBO and FMBO exhibited faster rates and higher arsenic removal (up to 96%) than PE-FMBO. Maximum As(III) adsorption capacities ranged from 4.76 to 5.75 mg/g for PE-FMBO, 7.2 to 12.0 mg/g for PET-FMBO, and up to 20.8 mg/g for FMBO, while capacities for As(V) ranged from 5.20 to 5.60 mg/g, 7.63 to 18.4 mg/g, and up to 46.2 mg/g, respectively. The results of the Dubinin–Radushkevich isotherm model, with free energy (E_a_) values exceeding 16 kJ/mol, suggest chemisorption is the dominant mechanism, which is supported by the kinetics data. Given the effective removal of As(III), chemisorption likely proceeds through ligand exchange during the Mn oxide-mediated oxidation of As(III) and complexation with hydroxyl groups on the nanocomposite. These findings highlight the strong potential of Fe–Mn polymer nanocomposites, particularly PET-FMBO, for efficient arsenic removal during practical water treatment applications.

## 1. Introduction

The increasing challenges of ensuring a safe drinking water supply, exacerbated by climate change, underscore the critical importance of groundwater, which is essential for global water supply, irrigation, and ecosystem support, especially in regions with limited surface water. However, natural geological processes, particularly the weathering of arsenic-containing minerals and the reduction of arsenic-bearing sediments, have led to significant groundwater contamination in many parts of the world [1,2]. These geochemical processes are responsible for the release of arsenic into aquifers, where it poses a serious threat to drinking water supplies.

Among these pollutants, arsenic is one of the most concerning due to its prevalence in groundwater, where it is mobilized primarily through natural geochemical reactions such as desorption, dissolution, and reductive dissolution of arsenic-bearing minerals [3]. While anthropogenic activities, such as mining, industrial processes, and the use of arsenic-based pesticides, contribute to arsenic contamination, geogenic sources account for the majority of arsenic found in groundwater worldwide [4,5]. Approximately 230 million people globally are affected by arsenic-contaminated groundwater [6,7], with the highest concentrations found in regions where natural geochemical processes dominate arsenic mobilization, such as South and Southeast Asia, parts of Latin America, and some areas of Europe [8,9,10,11,12]. It is well established that chronic exposure to arsenic through drinking water can lead to severe health issues, including skin lesions, cardiovascular diseases, neurological disorders, and cancer [13]. To decrease risk and protect public health, the World Health Organization (WHO) has recommended a maximum allowable concentration of arsenic in drinking water of 10 μg/L. Many countries in Europe have adopted this guideline or have established their own regulations based on similar thresholds to ensure the safety of drinking water for their populations [14].

In groundwater, arsenic predominantly exists in two inorganic forms: As(III) and As(V), with As(III) being more toxic and mobile, especially in deeper, reducing aquifers where geochemical conditions favor its release. This presents a challenge for water treatment systems, which often require pre-oxidation steps to convert As(III) to As(V) for more effective removal. The natural geochemistry of arsenic compounds and their speciation in groundwater complicate treatment processes, increasing operational costs and necessitating innovative, cost-effective solutions that can simultaneously remove both forms of arsenic.

Adsorption has emerged as one of the most effective methods for arsenic removal due to its simplicity, cost-effectiveness, and adaptability to various water treatment systems. A range of natural and synthetic adsorbents have been developed, with iron-based and carbon-based materials showing particular promise for addressing arsenic contamination [15,16,17]. Recent advancements in nanotechnology have further improved adsorption capacities by exploiting the high surface area and reactivity of nanomaterials, such as binary metal oxides [18,19,20]. Among these, Fe–Mn binary oxide (FMBO) has been widely studied due to its combined oxidation and adsorption capabilities, making it a highly efficient material for removing As(III) and As(V) from water [21,22,23].

However, practical challenges such as nanoparticle aggregation, separation, and reusability limit the application of FMBO in fixed-bed or flow-through systems. One approach to maximize the benefits of FMBO nanoparticles for arsenic removal and overcome these drawbacks is to immobilize or load FMBO onto granular carriers such as GAC, diatomite, graphene oxide, etc. [24,25,26,27]. The use of non-toxic natural polymers such as chitosan and starch as carriers for FMBO has also been proposed as a more environmentally friendly approach [28,29,30]. However, their stability during water treatment, potential for scalability, and cost-effectiveness of such composite materials must be considered for their potential commercialization and widespread application.

Synthetic polymers, particularly polyethylene (PE) and polyethylene terephthalate (PET) are widely used due to their high chemical, mechanical, and thermal stability, making them suitable for large-scale applications. Although their inert surfaces limit arsenic adsorption, their use as carriers for FMBO nanoparticles represents an innovative and sustainable solution for arsenic removal in water treatment [31,32,33]. For example, only 14% of PET waste is currently recycled, leading to products (fibers, granules) with low added value but with a remarkable negative impact on living organisms and the environment [34,35]. Such materials, especially those in the form of granules, could be beneficial for application in filtration systems in water treatment as well as alternative recycling methods to derive higher-value products from such waste, in accordance with the principles of the circular economy [33,36,37].

The aim of this study is to evaluate the adsorption performance of innovative Fe–Mn binary oxide (FMBO) nanocomposites, synthesized by coating polyethylene (PE) and polyethylene terephthalate (PET) with FMBO, for the removal of As(III) and As(V) from water. This novel approach combines the oxidation and adsorption capabilities of nanoscale FMBO with the stability, mechanical strength, and hydraulic properties of PE and PET, enabling the development of robust materials suitable for fixed-bed and continuous filtration systems. The study involves a comprehensive physicochemical characterization of the synthesized FMBO nanocomposites and their precursors, followed by a detailed evaluation of their adsorption behavior. Various kinetic and isotherm models were employed to model the adsorption mechanisms of As(III) and As(V) on the nanocomposites, providing insights into the adsorption dynamics and equilibrium characteristics and establishing the feasibility of these materials for practical water treatment applications.

## 2. Materials and Methods

### 2.1. Materials

Commercial granular low-density polyethylene and commercial polyethylene terephthalate were purchased from Sigma Aldrich. FeSO_4_ 7H_2_O (Lach-Ner) and KMnO_4_ (Acros Organics, Geel, Belgium) were used for FMBO nanocomposite preparation. As_2_O_3_ and As_2_O_5_ (Sigma Aldrich, St. Louis, MO, USA) were used to prepare stock solutions of As(III) and As(V) by dissolving them in deionized water.

### 2.2. Synthesis of FMBO and FMBO Nanocomposites

The FMBO (with an Fe:Mn molar ratio of 3:1) utilized in this study was synthesized according to a method previously reported by Nikić et al. [22]. The PE and PET were coated with Fe–Mn binary oxide (Fe/Mn molar ratio 3:1) as follows: 10 g of polymers were mixed with 200 mL of a binary solution containing KMnO_4_ (0.015M) and FeSO_4_ (0.225 M). After stirring for 24 h at room temperature, the coated polymers were washed with distilled water and transferred to a solution of 1 M NaOH, where they were stirred for another 24 h at room temperature. The resulting FMBO nanocomposite was rinsed with distilled water until a neutral pH was reached and then dried at room temperature. Both the FMBO nanocomposites and their precursors are presented in Figure S1 of the Supplementary Material.

### 2.3. FMBO Nanocomposites Characterization

The specific surface areas, pore sizes, and pore volumes of the adsorbents were obtained using the Auto-sorb iQ Surface Area Analyzer (Quantachrome Instruments, Boynton Beac, FL, USA). The Brunauer–Emmett–Teller (BET) method was used to determine the surface area, while the mesopore volumes were derived from the desorption isotherms using the Barrett–Joyner–Halenda (BJH) method. The Density Functional Theory (DFT) approach was employed to determine the pore size distribution, with the micropore volume determined using the t-plot method.

The X-ray diffraction (XRD) patterns of the adsorbents were recorded using a Rigaku MiniFlex 600 diffractometer (Tokyo, Japan) with Cu-Kα radiation (λ = 1.5418 Å), covering a 2θ range from 10° to 90°. The measurements were performed with a step size of 0.02° and a dwell time of 3 s per step.

Morphological structures and surface elemental compositions of the materials were examined via scanning electron microscopy (SEM) (TM3030, Hitachi High-Technologies, Tokyo, Japan) coupled with energy dispersive spectrometry (EDS) (Bruker Quantax 70 X-ray detector system, Bruker Nano GmbH, Berlin, Germany).

The functional groups present on the adsorbents were identified by Fourier transform infrared (FTIR) spectrometry (Nicolet iS20 FTIR spectrometer, Thermo Fisher Scientific, Waltham, MA, USA), operating in diffuse reflection mode with a resolution of 4 cm^−1^. The points of zero charge (pHpzc) of the adsorbents were determined by varying the pH value of 0.1 M NaNO_3_ solutions in the range from 2 to 10, according to the method previously reported by Šolic et al. [38].

### 2.4. Arsenic Adsorption Experiments

Arsenic adsorption experiments were conducted in 40 mL glass bottles containing 20 mL of aqueous solution and 0.5 g of adsorbent. In the kinetics experiments, the initial concentrations of As(III)/As(V) were set at 0.2 mg/L, and the pH of the solution was adjusted to 6.0, 7.0, or 8.0 using 0.1 M HNO_3_ and/or NaOH. The suspensions were agitated on an orbital shaker for 24 h at 180 rpm and ambient temperature. Samples were collected at predetermined intervals (0.25, 0.5, 0.75, 1, 1.5, 2, 4, 7, 9, 12, 16, 24 h), and the remaining arsenic concentrations in the supernatant were measured by inductively coupled plasma mass spectrometry (ICP/MS). Adsorption isotherms were generated by varying the initial arsenic concentration within the range of 0.2–2 mg/L while keeping all other parameters constant, as in the kinetics test. All adsorption experiments were performed in triplicate, with error bars included to show the standard deviations of measurements.

To assess the reusability of the Fe–Mn polymer nanocomposite, three successive adsorption–desorption cycles were performed. For the adsorption test, 0.5 g of adsorbent was introduced into bottles containing 20 mL of arsenic solution (0.2 mg/L). After stirring for 4 h at pH 7.0 ± 0.2, the adsorbents were separated from the solution, and the remaining arsenic concentrations were analyzed using ICP/MS. The used adsorbent was gently rinsed with deionized water and then dispersed into 20 mL of regenerant solution, where it was shaken for 4 h.

Before selecting the optimal regenerant for Fe–Mn polymer nanocomposites, preliminary experiments were conducted to evaluate different regeneration solutions, including 0.1 M NaOH, 0.5 M NaOH, and a mixture of NaOH, NaOCl, and NaCl. Although typical regeneration of Fe–Mn-based adsorbents involves a mixture of NaOH, NaOCl, and NaCl to desorb arsenic and reoxidize Mn(II) to Mn(IV), our experimental results showed that 0.1 M NaOH was sufficient for desorption, providing a simpler and effective regeneration method for the materials used in this study [39]. As a result, 0.1 M NaOH was selected for arsenic desorption from the Fe–Mn polymer nanocomposite. The amount of desorbed arsenic was determined by measuring the arsenic concentration in the regenerant solution. Before starting the next adsorption–desorption cycle, the adsorbent was thoroughly rinsed until a neutral pH (7) was achieved.

### 2.5. Adsorption Kinetics and Isotherms Modelling

#### 2.5.1. Adsorption Kinetics

To investigate the adsorbate uptake rate and gain insight into potential mechanisms or reaction pathways of the As(III) and As(V) adsorption on FMBO nanocomposite, three kinetic models were employed. These models include the Lagergren pseudo-first-order, pseudo-second-order, and Elovich models. The non-linear equations and parameters of these kinetic models are detailed in Table S1.

#### 2.5.2. Adsorption Isotherms

To acquire data regarding the maximum adsorption capacity of the adsorbents and to gain a deeper understanding of the adsorption mechanisms, the experimental equilibrium data were analyzed using the Freundlich, Langmuir, Temkin, and Dubinin–Radushkevic (D–R) isotherm models. Table S1 presents the non-linear mathematical expressions, parameters, and significant assumptions of these models. Additionally, it provides equations for the separation factor (also referred to as the equilibrium parameter) and adsorption activation energy, which are crucial aspects of the Langmuir and D–R isotherms.

### 2.6. Analytical Methods

pH measurements were carried out using an InoLab pH/ION 735 instrument (WTW GmbH, Weilheim, Germany). Arsenic, iron, and manganese concentrations were determined by ICP/MS (Agilent Technologies 7700xSeries ICP-MS, Tokyo, Japan). Method detection limits for the arsenic, iron, and manganese were 0.001 mg/L.

## 3. Results and Discussion

### 3.1. FMBO Nanocomposite Characterization

**Figure 1:**
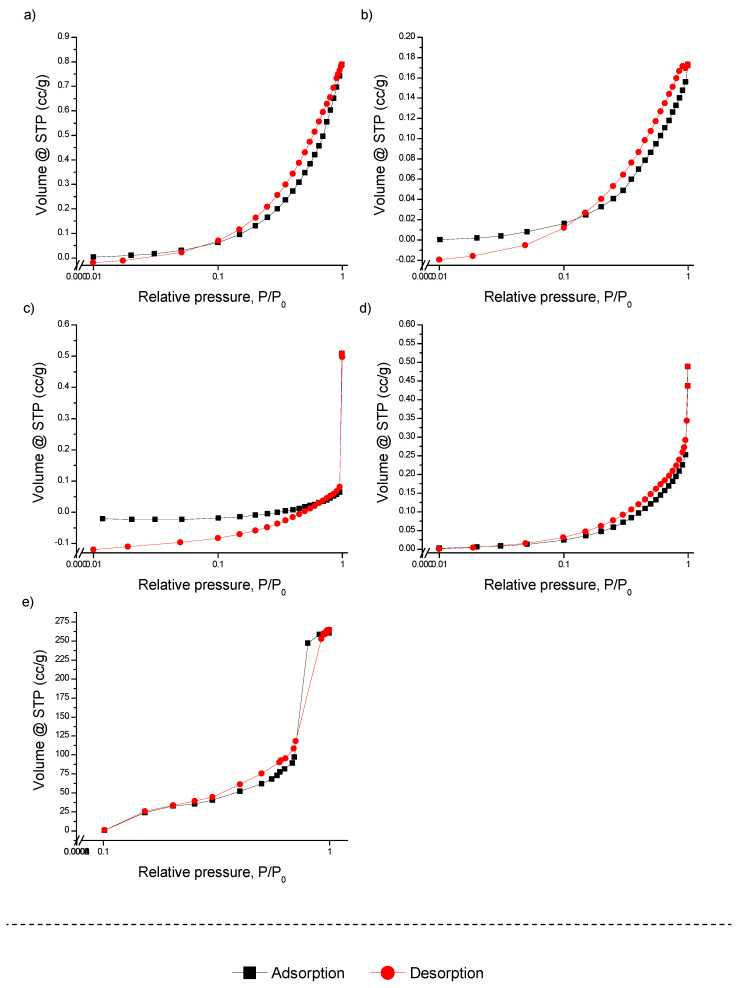
N_2_ adsorption–desorption isotherm of (**a**) PE, (**b**) PE-FMBO, (**c**) PET, (**d**) PET-FMBO, and (**e**) FMBO.

The nitrogen adsorption–desorption isotherms (BET), along with the pore size distribution determined by BJH and DFT calculations, are presented in Figure 1, Figures S2 and S3 for both unmodified and modified FMBO nanocomposites. As shown in Figure 1, all isotherms for the modified polymers are of type III with H3 hysteresis, which is relatively uncommon and can be attributed to polymer structures with non-rigid aggregates of plate-like particles and slit-shaped pores. This behavior suggests that the polymers maintain their structural characteristics even after modification with FMBO, and the lack of significant changes in the isotherm shapes indicates that the surface modification did not introduce major new porosity or alter the existing polymer network. Such a result implies that the FMBO coating forms a thin layer on the polymer substrates rather than infiltrating deeply into the porous structure.

In contrast to polymers, the N_2_ adsorption–desorption isotherm of FMBO alone aligns with type IV isotherms, exhibiting H3 hysteresis loops typical of mesoporous materials [40]. This is indicative of a well-developed mesoporous structure with a random pore distribution and interconnected pores, making FMBO a highly efficient arsenic adsorbent material, as previously confirmed [22].

The calculated BET surface areas of the unmodified granular polymers, PE and PET, were 0.248 and 0.325 m^2^/g, respectively. After modification with Fe–Mn binary oxide, the specific surface areas of the polymers did not change significantly (Table 1), with PE showing only a 0.7-fold increase and minimal changes for PET. This minimal change in surface area supports the hypothesis that the FMBO layer does not significantly alter the intrinsic surface properties of the polymers. The polymer substrates themselves, being relatively non-porous, do not contribute significantly to surface area but serve as stable, easy-to-handle carriers for the FMBO active layer.

Interestingly, although mesopore and micropore volumes in the polymers were insignificant, BJH and DFT analyses demonstrated that mesopores dominated the pore structure in all materials, with a very small proportion of macropores (around 1%) (Figures S2 and S3). This observation is important, as mesoporous materials are particularly suited for applications involving the removal of small contaminants, such as arsenic, because their pore sizes facilitate both the rapid diffusion of contaminants and strong adsorption interactions. For Fe–Mn binary oxide, the BJH and DFT pore size distribution showed an average pore size ranging from 10 to 100 nm, with the majority of the pore volume concentrated around 10 nm (Figures S2 and S3).

Based on these results, it can be inferred that FMBO forms a thin, uniform layer on the polymer surface without significantly altering the overall porous structure. While this maintains the polymer’s structural integrity, the limited increase in surface area suggests that further improvement in adsorption capacity could be achieved by increasing the surface area, which would provide more active sites for arsenic. Optimizing the modification process to create a more porous FMBO layer or using polymers with higher initial surface areas could enhance performance. Nonetheless, the current approach strikes a balance between maintaining mechanical properties and enhancing adsorption efficiency while also being cost-effective for large-scale production.

The morphology of unmodified and FMBO nanocomposite polymers was investigated by scanning electron microscopy (SEM), and corresponding SEM images are given in Figure 1.

**Figure 2:**
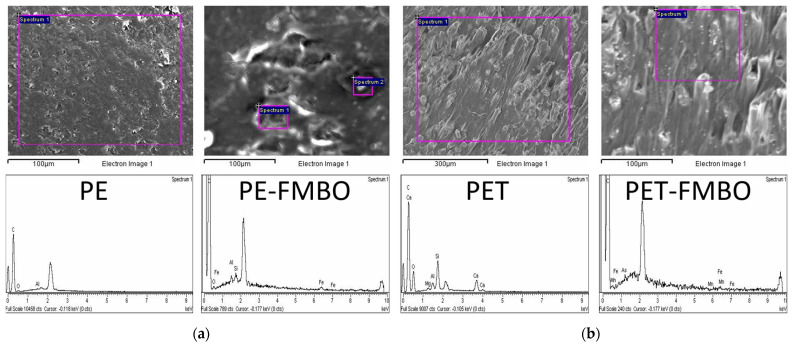
SEM/EDS of unmodified polymer and FMBO nanocomposite (**a**) PE and PE-FMBO; (**b**) PET and PET-FMBO.

SEM micrographs revealed that PE was characterized by relatively smooth surfaces (Figure 2a), while PET exhibited an uneven surface texture, especially on the sides of the particles (Figure 2b). After modification with Fe–Mn binary oxide, the surfaces of both composites, PE-FMBO and PET-FMBO, were covered with many agglomerated particles of irregular shape, indicating the presence of an amorphous layer formed by a large number of Fe–Mn binary oxide nanoparticles [22]. These observations lead to the assumption that during the synthesis, the polymer surface was well-distributed with Fe–Mn binary oxide particles, which resulted in modified materials with more active adsorption sites. EDS analysis was further employed to ascertain the elemental composition of the polymers and FMBO nanocomposites, and the results are presented in Figure 1 and Table 2.

As seen in Figure 1 and Table 2, the main EDS-detectable elements present in all polymer materials are carbon (C) and oxygen (except for PE), which are associated with their polymer structure (there is, of course, also hydrogen present, but it cannot be detected by EDS). Upon modification of PE with FMBO, additional peaks for Fe (5.78%), Mn (2.02%), and O (5.80%) appear, indicating the presence of FMBO. Furthermore, the carbon content decreases from 100% to 78%. Similarly, after modifying PET with FMBO, the appearance of Fe and Mn peaks, along with an increase in oxygen content from 25.5% to 29.2%, suggests the formation of Fe and Mn oxides.

The surface analysis of PE-FMBO and PET-FMBO revealed the presence of Fe and Mn in a Fe:Mn molar ratio of 2.86:1 and 3.12, respectively, which is consistent with the expected Fe:Mn molar ratio of 3:1.

**Figure 3:**
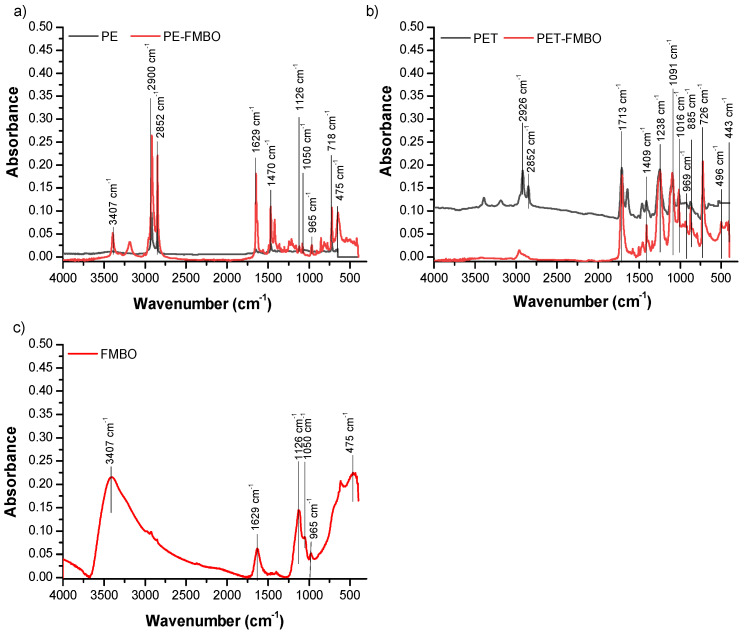
FTIR spectra of unmodified polymer and FMBO nanocomposite (**a**) PE and PE-FMBO, (**b**) PET and PET-FMBO, and (**c**) FMBO.

To further identify the changes in the polymer surface after modification with FMBO, FTIR analysis was employed (Figure 3).

It was observed that all spectra exhibited bands at 3407 cm^−1^ and 1629 cm^−1^, attributed to the O–H stretching vibration and O–H bending vibrations of the hydroxyl group, indicating the presence of physically adsorbed water molecules [41]. Additionally, in FTIR spectra of PE, characteristic peaks for this polymer were observed at 2918 cm^−1^ and 2849 cm^−1^ (C–H stretching vibration), 1467 cm^−1^ and 1383 cm^−1^ (C–H bending vibration), and 721 cm^−1^ (rocking vibration of the –CH_2_– group) (Figure 3a) [42]. Characteristic bands for polyethylene terephthalate were detected at 1713 cm^−1^ (C=O stretching of the keto group), 1409 cm^−1^ (C–O stretching and O–H group deformation), 1238 cm^−1^ (terephthalate group –OOCC6H4–COO), 1091 cm^−1^ (vibrations of the ester C–O bond from poly(ethylene terephthalate)), 1014 cm^−1^ assigned for in-plane vibration of benzene, 930 cm^−1^ for O–H stretching, 872 cm^−1^ for C–H stretching, and 723 cm^−1^ ethyl group bending [37,43] (Figure 3b). In the FTIR spectra of FMBO (Figure 3c), bands at 1112 cm^−1^, 965 cm^−1^, 1050 cm^−1^, and 1126 cm^−1^ were observed, originating from the deformation vibrations of the hydroxyl group (–OH), responsible for the formation of internal spherical surface complexes [44,45]. The sharp peak observed at 457 cm^−1^ originates from the valence Fe–O or Mn–O vibration [24,45].

After modification of polymers with Fe–Mn binary oxides, new peaks appear in the range of 419–775 cm^−1,^ which can be attributed to vibrations of Fe–O and Mn–O [27]. Furthermore, new peaks detected in the upper range (965–1244 cm^−1^) can be related to the vibration of the –OH group on the surface of FMBO nanocomposites. All of these changes confirmed the surface coating of the polymers with FMBO.

**Figure 4:**
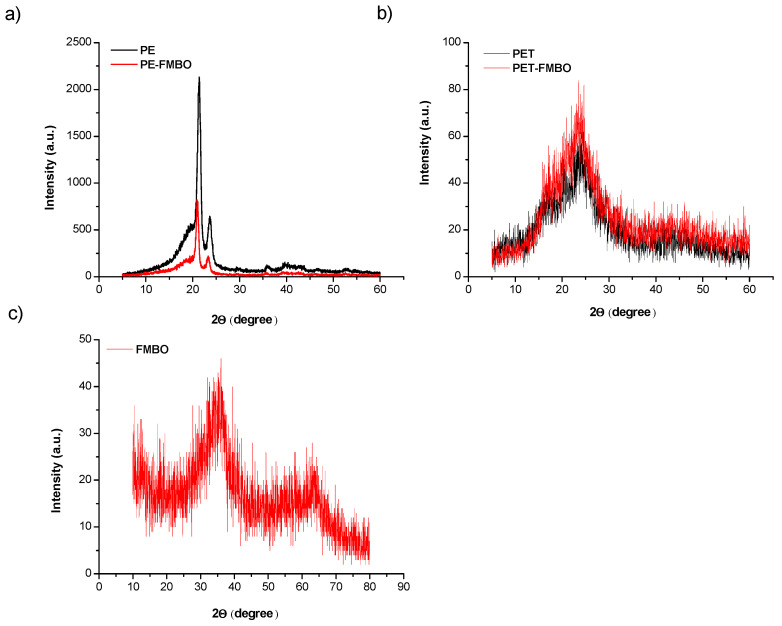
XRD pattern of unmodified polymer and FMBO nanocomposite (**a**) PE and PE-FMBO, (**b**) PET and PET-FMBO, and (**c**) FMBO.

X-ray diffraction (XRD) is commonly used to study the crystallinity of materials and to identify phase content. The XRD pattern of PE typically reveals its semi-crystalline nature, characterized by both crystalline and amorphous regions. The crystalline regions produce sharp diffraction peaks, commonly observed at 2θ values around 21° and 24° [27], corresponding to the (110) and (200) planes of the orthorhombic crystal structure. In contrast, the amorphous regions are represented by a broad hump, indicating the disordered portions of the polymer (Figure 4a). After modification with FMBO nanoparticles, the XRD analysis of the PE-FMBO composite showed a reduction in both the sharp crystalline peaks and the broad hump associated with amorphous regions (Figure 4a). The less pronounced crystalline peaks suggest a decrease in overall crystallinity, likely caused by the disruption of polyethylene’s ordered regions due to the incorporation of FMBO nanoparticles. The diminished broad hump indicates potential alterations in the polymer matrix, where interactions between nanoparticles and polymer chains have affected the structure of the disordered regions. These changes suggest that the nanoparticles influence both the crystalline and amorphous phases of the polymer, resulting in altered structural properties.

XRD analysis of PET typically reveals its semi-crystalline structure, with distinct crystalline peaks observed between 2θ = 16°–26°, corresponding to the ordered regions of the polymer. A broad, amorphous region is also present in the pattern (Figure 4b). After modification with FMBO nanoparticles, the XRD pattern of PET-FMBO composites shows an increase in the intensity of both the crystalline peaks and the amorphous regions. This suggests that the FMBO nanoparticles may act as nucleating agents, promoting the growth of crystalline regions. The enhanced crystallinity could indicate improved structural ordering of the polymer chains caused by the presence of FMBO nanoparticles, while the increased intensity in the amorphous regions may reflect changes in the polymer matrix due to the incorporation of the nanoparticles.

XRD analysis of Fe–Mn binary oxides indicates that both iron oxide and manganese oxide are present in an amorphous form, as no significant crystalline peaks were identified in the materials (Figure 4c). This amorphous structure likely contributes to the large specific surface area observed for the binary oxides (Table 1). Amorphous iron oxides are known for their high specific surface area and abundance of surface-active sites, which tend to decrease significantly when crystalline phases form. Additionally, two broad peaks were observed at 2θ = 34.6° and 61.2°, which resemble those of ferrihydrite, with comparable peaks at 34.4° and 62.1° (d-values of 0.260 and 0.149 nm, respectively). Since the XRD pattern of ferrihydrite similarly shows two broad peaks at 34.4° and 62.1° (with d-values of 0.260 and 0.149 nm, respectively), it can be suggested that the phase structure of the Fe–Mn binary oxides is comparable to that of ferrihydrite [46]. This similarity may also explain the large specific surface areas of Fe–Mn binary oxides, as ferrihydrites are known to have specific surface areas in the range of 200–300 m^2^/g [22].

The isoelectric point (pHpzc) of an adsorbent is a crucial factor that affects its behavior in water with different acidity levels (pH). When the pH is lower than the pHpzc, the surface of the adsorbent becomes positively charged, which helps it attract negatively charged anions. However, when the pH is higher than the pHpzc, the sorbent’s ability to bind with anions gradually decreases. According to Figure S4 in the Supplementary Material, the point of zero charge obtained for unmodified granular polymers, PE and PET, were pHpzc = 4.61 and pHpzc = 5.58, and these values were consistent with the findings of other authors [47]. After modifying the polymers with FMBO, higher pHpzc values were observed, with pHpzc values of 6.62 and 6.16 for PE-FMBO and PET-FMBO, respectively. The pHpzc of FMBO is 6.54 [22], which is very close to the values obtained for the FMBO nanocomposites, once again confirming the successful surface coating of the polymers.

### 3.2. Arsenic Adsorption Performance

To investigate the adsorption performance of synthesized FMBO nanocomposites, kinetic and equilibrium experiments were conducted. These experiments evaluated the arsenic adsorption performance of novel FMBO nanocomposites, PE-FMBO and PE-FMBO. The unmodified polymers were not investigated in this manner, as preliminary experiments had shown the efficiency of As(III) and As(V) removal on PE and PET was below 5%, as expected (Figure S3, Supplementary Material). Instead, FMBO itself is used to compare the nanocomposites investigated.

#### 3.2.1. Adsorption Kinetics

**Figure 5:**
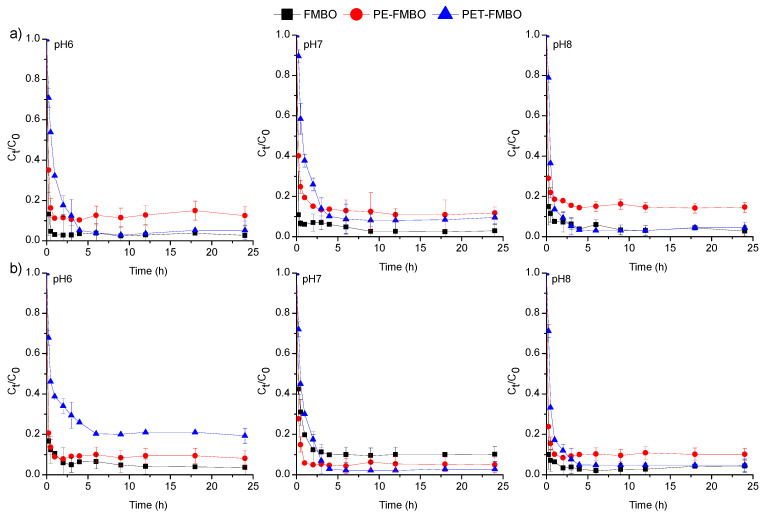
Adsorption of (**a**) As(III) and (**b**) on FMBO, PE-FMBO, and PET-FMBO as a function of contact time (m = 0.5 g, V = 20 mL (0.1 M NaNO_3_), C_0_ = 0.2 mg/L, pH = 6–8.

The effect of contact time on the adsorption of As(III) and As(V) onto the PE-FMBO and PET-FMBO was investigated over 24 h at three pHs (pH 6, 7, and 8). The obtained results are presented as changes in the concentration of arsenic (C_t_/C_o_) as a function of contact time (Figure 5).

**Figure 6:**
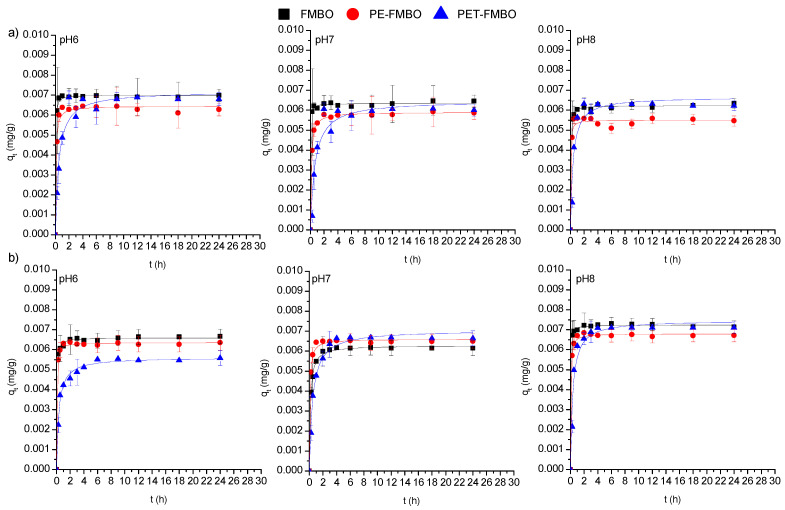
Non-linear regressions of the pseudo-second-order kinetics model for (**a**) As(III) and (**b**) As(V) adsorption on FMBO, PE-FMBO, and PET-FMBO at pH 6, 7, and 8.

The adsorption of As(III) and As(V) on FMBO and PET-FMBO was initially rapid and reached equilibrium after 1–2 h at all investigated pH values. After this period, the arsenic concentrations remained consistently stable, averaging 95.3–96.7% of As(III) and 89.6–96.8% of As(V) removal using FMBO and 88.1–95.4% of As(III) and 76.2–95.1% of As(V) removal on PET-FMBO, depending on the pH. The adsorption of As(III) and As(V) on PE-FMBO was also fast, but equilibrium was reached within 4 h at all three pH values. Throughout the observed time period, the degree of As(III) and As(V) removal was 82.6–88.2% and 89.9–94.9%, respectively, depending on pH. The longer contact time required for established equilibrium for As(III) and As(V) on PE-FMBO can be attributed to the gradual saturation of adsorption sites as a result of fewer active sites on its surface and a lower specific surface area. However, the relatively short time required for equilibrium in all investigated cases suggests that the adsorption of both arsenic species on these materials is primarily influenced by interactions occurring on the surface of the adsorbate/adsorbent [19,38]. Studying the kinetics of the adsorption process using suitable kinetic models provides insights into the rate and extent of transfer, as well as the degree of accumulation of the adsorbate on the surface of the given adsorbent. Additionally, kinetic modeling can reveal valuable insights into adsorption mechanisms and the steps or processes governing the rate of adsorption. Consequently, to elucidate the mechanism of arsenic adsorption on FMBO nanocomposite, the most common kinetic models, pseudo-first and pseudo-second-order, and the Elovich model, were employed to fit the kinetic data (Figure 6, Table S2). Based on the obtained results, the pseudo-second-order model provided a more accurate description in all cases, exhibiting higher correlation coefficients for As(III) (R2 = 0.932–0.998) and As(V) adsorption (0.964–0.999) compared to the pseudo-first-order and Elovich models. This implies that the major mechanism beyond the kinetic adsorption process of both As(III) and As(V) is the chemisorption-formation of chemical bonds [27]. The same findings were documented by Xu et al. (2024), who examined the adsorption kinetics of As(III) and As(V) on Fe–Mn–O/bamboo monolith (MB) composites, suggesting that chemisorption controls the adsorption of both arsenic species on Fe–Mn–O/MB composites [48].

The rate constants (k_2_) were significantly higher for As(III) adsorption on FMBO (5729–9092 g/mg h) and PET-FMBO (1041–2178 g/mg h), compared to the k_2_ values obtained for adsorption on PE-FMBO (234–402 g/mg h) at all three pHs. The same trend was observed for As(V) adsorption, where k_2_ values were in the range of 2073–6735 g/mg for FMBO, 1054–3425 g/mg h for PE-FMBO, and 290–537 g/mg h for PET-FMBO. In addition, it was noticed that in all cases, the initial adsorption rate (h) was lower than k_2_, confirming that the adsorption of both arsenic species on FMBO, PE-FMBO, and PET-FMBO was faster at the beginning of the adsorption process. This phenomenon can be explained by the basic mechanisms of adsorption and the hydrodynamics of the system. At the beginning of the process, the number of adsorption sites and the concentration of arsenic in the solution are at their maximum, resulting in the maximum driving force of the adsorption process. Additionally, mixing provides the energy necessary for the transport of arsenic through the liquid film to the active sites on the adsorbent, thereby reducing the resistance during mass transfer between the liquid phase and the adsorbent. Consequently, all three factors mentioned are the presence of a large number of active sites, a significant driving force, and reduced resistance to mass transfer due to mixing-promoted adsorption [49]. However, over time, as arsenic accumulates on the surface of the adsorbent and the resulting concentration gradient decreases, the rate of arsenic sorption gradually decreases, reaching a state of equilibrium where the rate of adsorption equals the rate of desorption.

The suitability of the pseudo-second-order model for describing the adsorption of As(III) and As(V) on the investigated materials was also confirmed through the best fit of the experimentally determined (q_e,exp_) and theoretically determined values of the adsorption capacity (q_e_). The results given in Table S2 suggest that the adsorption capacity of FMBO nanocomposite, including FMBO for As(III) and As(V), remained unchanged when the pH of the solution varied from 6 to 8, indicating that charge interactions play an insignificant role in arsenic adsorption (Section 3.3). This indicates that the materials could be highly suitable for real groundwater applications.

#### 3.2.2. Adsorption Isotherms

The investigation of the adsorption affinity of the synthesized nanocomposite for As(III) and As(V) was conducted through batch experiments under equilibrium conditions. The obtained experimental data were modeled using different isotherm models, including the Freundlich, Langmuir, Temkin, and Dubinin–Radushkevich isotherms, and the corresponding parameters of the applied models are shown in Table 3.

**Figure 7:**
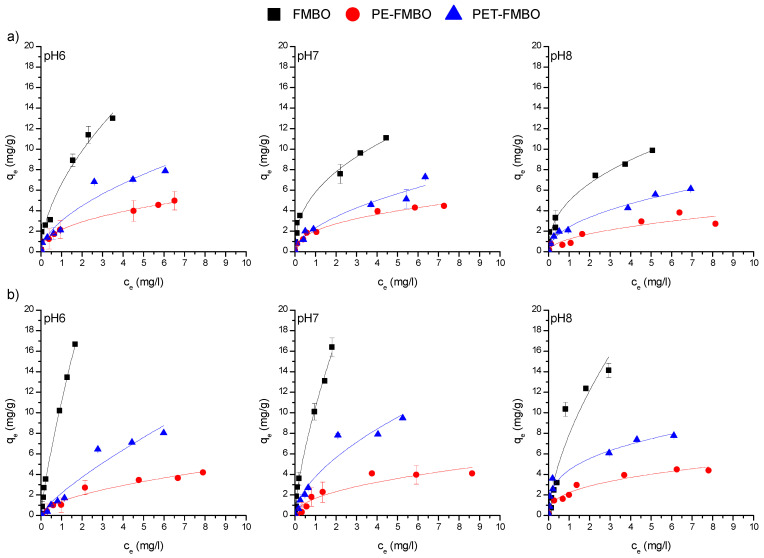
Freundlich adsorption isotherms of (**a**) As(III) and (**b**) As(V) on FMBO, PE-FMBO and PET-FMBO (m = 0.5 g, V = 20 mL (0.1 M NaNO_3_), C_0_ = 0.1–10 mg/L, pH = 6–8).

Analysis of the coefficients of determination (R^2^) indicates that, with the exception of the Temkin model, the models all showed good agreement with the experimental data (Table 3). Nonetheless, the Freundlich model demonstrated a slightly better fit for As(III) and As(V) adsorption on PE-FMBO, PET-FMBO, and FMBO at all investigated pH (Figure 7 and Table 3).

The better fit of the Freundlich model in most of the observed cases can be attributed to the heterogeneous surface of the FMBO nanocomposite adsorbents and multilayer adsorption [50]. This means that the affinities of adsorbate towards the various surfaces are not uniform and that adsorbate molecules are likely interacting with different sites on the surface of the adsorbent material, leading to the formation of multiple layers of adsorbate molecules. This could be explained by varied surface characteristics, such as the different particle shapes and sizes observed in the SEM images of the materials (Figure 1). The observed non-uniformity of the surface provides a variety of adsorption sites, facilitating the formation of multiple layers of adsorbate molecules and resulting in a better fit for the Freundlich model. In addition, the uptake of As(III) by the PET-FMBO, PE-FMBO, and FMBO is a complex process involving not only adsorption but also redox reactions occurring on the surface, which cannot be explained by the Langmuir model [51], as the Langmuir model assumes monolayer adsorption on homogeneous sites with constant adsorption energy, where adsorption and desorption rates are presumed to be equal. Additionally, the Langmuir model does not account for the possibility of the oxidation of dissolved species resulting from interactions with the surface [52].

The Freundlich constant (K_f_), as a measure of adsorption affinity at all three pH for As(III) and As(V) adsorption, was in the following order: FMBO > PET-FMBO > PE-FMBO. The K_f_ values were consistent with the q_max_ values obtained by the Langmuir model.

The maximum adsorption capacities (q_max_) of As(III) onto PE-FMBO, PET-FMBO, and FMBO in this work obtained by the Langmuir model were in the range 4.76–5.75 mg/g, 7.2–12.0 mg/g and 11.0–20.8 mg/g, respectively, and for As(V) were 5.20–5.60, 7.63–18.4 mg/g, and 22.4–46.2 mg/g, respectively. Despite the decrease in arsenic adsorption capacities on both FMBO nanocomposites and FMBO as pH increases from pH 6 to pH 8, they still exhibit good performance.

As seen in Table 4, the FMBO nanocomposites synthesized in our study show competitive adsorption capacities for both As(III) and As(V) when compared to other adsorbents. For example, the GAC-FMBO composite demonstrated adsorption capacities of 2.87 mg/g for As(III) and 2.30 mg/g for As(V) at pH 7 [28], while the chitosan/Fe–Mn nanofibrous composite achieved a capacity of 4.59 mg/g for As(III) at pH 6.5 [51]. These values highlight the moderate adsorption capabilities of the previous materials.

In contrast, the PET-FMBO nanocomposite developed in our study exhibited significantly higher adsorption capacities of 8.74 mg/g for As(III) and 13.3 mg/g for As(V), demonstrating the enhanced performance of the FMBO nanocomposites synthesized in this work. Similarly, the PE–FMBO nanocomposite showed an adsorption capacity of 5.29 mg/g for As(III) and 5.37 mg/g for As(V), further emphasizing the utility of polymer-supported FMBO materials in arsenic removal applications. The biochar impregnated with Fe–Mn oxides (FMBC) reported by Lin et al. [53] achieved an adsorption capacity of 8.25 mg/g for As(III), which aligns with our PET–FMBO results, although our composite outperforms FMBC in terms of As(V) adsorption capacity.

**Table 4:** Comparison of maximum arsenic sorption capacities for Fe–Mn based adsorbents.

Adsorbent	Initial As Concentration (mg/L)	pH	q_max_ As(III) (mg/g)	q_max_ As(V) (mg/g)	Reference
GAC-FMBO	0.1–1	7	2.87	2.30	[28]
Chitosan/Fe–Mn nanofibrous composite	0.06–2.3	6.5	4.59	-	[54]
Chitosan-FMBO	0.1–1	7	3.91	3.89	[55]
Biochar (BC) impregnated with Fe–Mn binary oxides (FMBC)	0.2–50		8.25	-	[53]
D201 modified with FMBO	1–50	7	44.9	13.7	[39]
Diatomite coated with Fe–Mn binary oxide	0.05–50	7	1.68	-	[24]
Magnetite coated with FMBO	0.2–50	7	55.9	54.1	[56]
Fe–Mn binary oxide	0.2–50	7	46.9	57.6	[22]
PE-FMBO	0.1–10	7	5.29	5.37	This study
PET-FMBO	0.1–10	7	8.74	13.3	

On the other hand, the commercial polystyrene anion exchanger D201 modified with FMBO (D201-Fe/Mn) exhibited significantly higher adsorption capacities of 44.9 mg/g for As(III) and 13.7 mg/g for As(V) [39]. While this product demonstrates superior performance in terms of adsorption capacity, it is important to note that D201 is a commercial product optimized for maximum adsorption. In contrast, our study focuses on balancing adsorption efficiency with practical factors such as ease of regeneration, cost-effectiveness, and environmental considerations.

The higher values of q_max_ for FMBO when used without polymer support or with magnetite nanoparticles (magnetite coated with FMBO) with adsorption capacities of 55.9 mg/g for As(III) and 54.1 mg/g for As(V) [56], suggest that FMBO alone has superior adsorption and oxidation properties. This performance can be attributed to the larger surface area and higher Fe and Mn contents. However, it is well-known that using nano-structured FMBO alone can result in material aggregation and complex challenges in material handling, which limits its practical applicability in large-scale water treatment systems.

By incorporating FMBO into polymer matrices, as demonstrated with our PE-FMBO and PET-FMBO nanocomposites, we address these limitations. Although the adsorption capacities are somewhat lower than pure FMBO, the nanocomposites exhibit much better hydraulic properties, stability, and ease of application, which are crucial for real-world applications. These improvements were also noted by Chang et al. (2009) and Li et al. (2012), who found that materials like diatomite and anion exchanger D201 modified with FMBO showed lower As(III) adsorption capacities compared to FMBO alone but with enhanced hydraulic and operational properties that made them more suitable for practical use [24,39]. Future work may focus on optimizing the balance between adsorption efficiency and real-world applicability by adjusting the polymer matrix or modifying surface treatment techniques.

The Freundlich constant, 1/n, is an indicator of adsorption intensity or the heterogeneity of the adsorbent surface, and in all cases, was less than 1 (0.32–0.79), indicating that the adsorption of As(III) and As(V) on FMBO nanocomposites is favorable and predominantly occurred via chemisorption [53,57]. This was in accordance with the RL (separation factor) values from the Langmuir model, which ranged from 0 to 1, reinforcing the notion of irreversible or favorable adsorption [58].

In the case of the Dubinin–Radushkevich model, the values and trends of theoretical adsorption capacity (q_d_) derived from this model were in agreement with the Freundlich and Langmuir models. The highest values for As(III) and As(V) at all three pHs were observed for FMBO, while PET-FMBO showed higher capacities for As(III) and As(V) over the PE-FMBO in all investigated conditions (Table 3). The free energy of adsorption (E_a_) for As(III) and As(V) on FMBO nanocomposites, including FMBO, were above 16 kJ/mol, suggesting that chemisorption may be the preferred adsorption mechanism of arsenic on these materials. Specifically, when E_a_ values lie in the range of 1–8 kJ/mol, adsorption is physical, while values in the range of 8–16 kJ/mol indicate an ion-exchange process. When E_a_ is larger than 16 kJ/mol, it indicates a chemisorption process [59,60,61,62,63].

The correlation coefficients for the Temkin isotherm were lower than the other applied models, ranging from R^2^ = 0.598 to 0.843 for As(III) adsorption on FMBO adsorbents and 0.554–0.920 for As(V) adsorption (Table 3). This model was, therefore, not considered adequate to describe the adsorption systems studied in this work.

### 3.3. Adsorption Mechanism

The adsorption experiments in this study were conducted within the range of real groundwater pH levels (6–8), where the surface of the FMBO composites remains overall negatively charged with some portion of the positive net charge. Therefore, the contribution of electrostatic interactions in the adsorption of anionic As(V) on these materials is expected to be minor or negligible. Similarly, the electrostatic contribution in the adsorption of As(III) is also expected to be minimal, as As(III) exists in a neutral form as H_3_AsO_3_ under these pH conditions [26,27]. As a result, another adsorption mechanism governs the uptake of As(III) and As(V), with electrostatic interactions playing an insignificant role.

**Figure 8:**
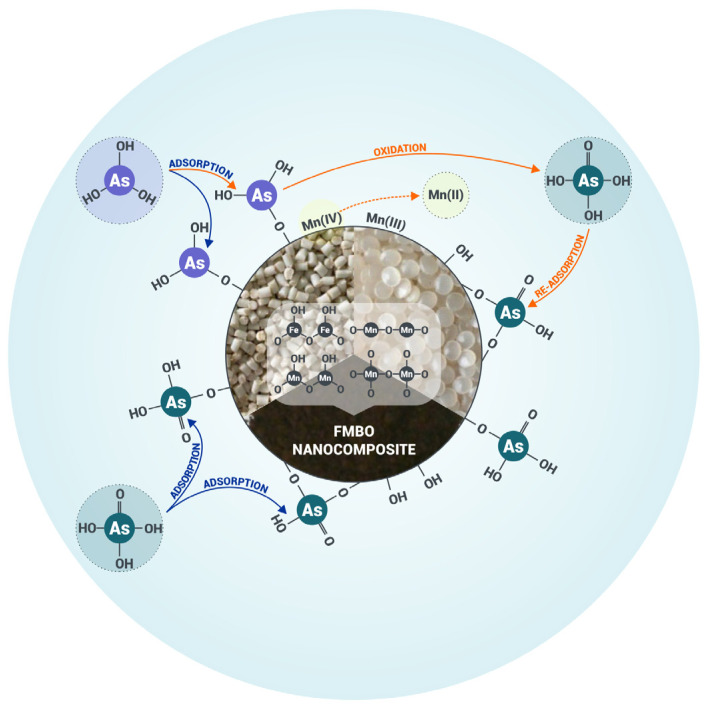
Proposed removal mechanism on Fe–Mn polymer nanocomposites.

The adsorption mechanism on Fe–Mn-based nanocomposites is driven by the dual roles of manganese and iron components (Figure 8). Manganese oxides (primarily Mn(IV)) are well-known for their oxidative capacity, facilitating the oxidation of As(III) to As(V) upon interaction with the adsorbent surface. The iron component of the composite, predominantly Fe(III), then adsorbs the resultant As(V) through surface complexation. This two-step process has been widely reported in studies where Fe–Mn-based sorbents were employed for arsenic removal [21,22,23,24,25,26,27,28,29,30,39].

Though XPS was not conducted in this study, previous research on similar adsorbents has demonstrated the role of Fe and Mn components in arsenic adsorption. XPS studies on Fe–Mn binary oxides, such as magnetic graphene oxide (MRGO)–FMBO, have confirmed that Fe(III) remains stable during the adsorption process, with Mn(IV) being reduced to Mn(III) or Mn(II) during the oxidation of As(III) [23,30,40]. This indicates that iron acts as the primary adsorbent for As(V), while MnO_2_ facilitates the oxidation of As(III). The higher Mn(III)/Mn(IV) ratio after adsorption indicates that MnO_2_ (predominantly Mn(IV)) facilitates the oxidation of As(III) to As(V), with Mn(III) accumulating as an intermediate product [40].

Additionally, the literature confirms that after As(III) adsorption, As(V) becomes the dominant species on FMBO, verifying that MnO_2_ efficiently oxidizes As(III) to As(V) [30,39,40]. Multiple studies note that the oxidation and adsorption processes are also accompanied by changes in the Mn valence state. A higher concentration of Mn^2+^ is thus observed after As(III) adsorption compared to As(V) adsorption (Table 5), supporting the oxidative transformation of As(III) to As(V) (Equation (1)) [21,22,23,24,25,26,27,28,29,30,39]. This release and potential re-adsorption of Mn^2+^ enhances the positive surface charge and promotes further adsorption of As(V) oxyanions [64,65].

MnO_2_ + H_3_AsO_3_ + H_3_O^+^→ Mn^2+^ + H_2_AsO_4_^−^ + 2H_2_O (1)

Furthermore, FTIR analysis in our study confirmed the presence of hydroxyl groups on PE-FMBO and PET-FMBO surfaces, which play a crucial role in forming stable complexes with both As(III) and As(V). The literature supports this observation with reports of hydroxyl groups facilitating arsenic adsorption through inner-sphere complexation [30,39,40]. EXAFS studies have also confirmed the formation of bidentate binuclear complexes, where arsenic bonds directly to Fe and Mn through ligand exchange.

In conclusion, our proposed adsorption mechanism involves two key processes: (1) direct adsorption of As(III) onto Fe and Mn through ligand exchange and (2) oxidation of As(III) to As(V), followed by the adsorption of As(V). Iron oxides serve as the primary adsorbent for As(V), while MnO_2_ plays a dual role as both an oxidant and adsorbent for As(III) (Figure 8). The existing literature strongly supports this dual mechanism, compensating for the lack of certain analytical techniques in this study.

### 3.4. Regeneration of Fe–Mn Polymer Nanocomposite and Reuse

**Figure 9:**
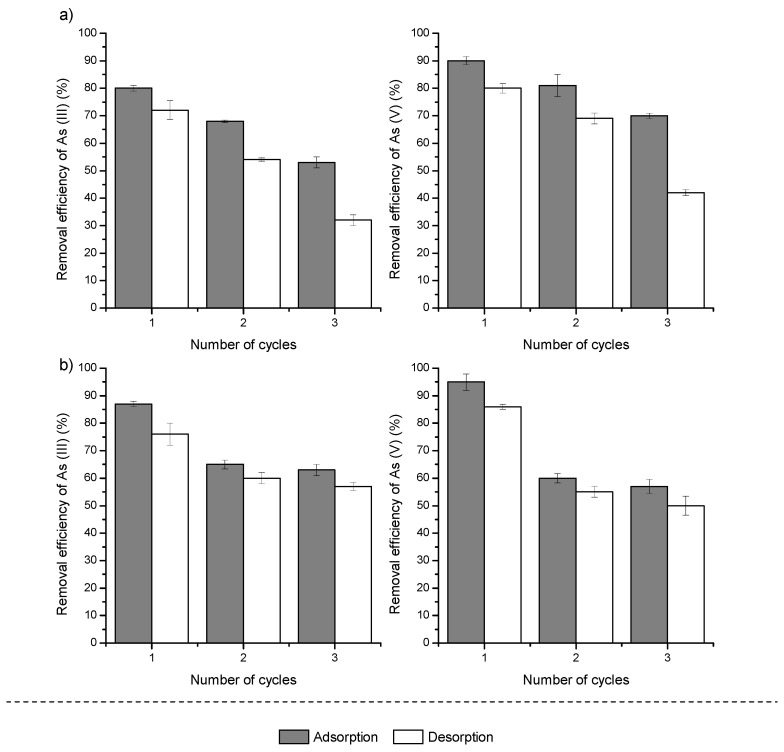
Regeneration and reuse of (**a**) PE-FMBO and (**b**) PET-FMBO after As(III) and As(V) adsorption. Adsorption conditions: Initial As(III)/As(V) 0.2 mg/L; sorbent dose 0.5 g; pH 7.0 ± 0.2. Desorption conditions: 0.1 M NaOH.

To evaluate the reusability of the Fe–Mn nanocomposite, three consecutive adsorption–regeneration cycles were performed (Figure 9).

Generally, as the number of regeneration cycles increases, the adsorption efficiency of PE-FMBO and PET-FMBO gradually decreases [28,55,56]. After the third adsorption/regeneration cycle, As(III) and As(V) removal by PE-FMBO decreased by 27% and 20%, respectively. Similarly, on PET-FMBO, after the third cycle, the removal efficiencies for As(III) and As(V) were 65% and 60%, respectively, compared to 87% and 95% removal of As(III) and As(V) after the first adsorption cycle (Figure 9).

The gradual decrease in adsorption capacities observed for both PE-FMBO and PET-FMBO after consecutive regeneration cycles can be attributed to several factors. One possible explanation is the progressive saturation or deactivation of active adsorption sites on the material’s surface. Over time, as arsenic species are adsorbed and desorbed, the structural integrity of the nanocomposites may degrade, leading to reduced availability of these sites. Additionally, repeated exposure to highly alkaline regenerant solutions could lead to slight alterations in surface chemistry or even physical degradation of the adsorbent, further contributing to the decline in performance.

Despite the observed decline, the materials still exhibit relatively high removal efficiencies, particularly after multiple cycles, suggesting that PE-FMBO and PET-FMBO are still viable for extended use, though with a reduced capacity. However, optimizing regeneration protocols or modifying the nanocomposites could help mitigate these effects and enhance their long-term reusability.

## 4. Conclusions

The successful modification of granular carriers, PET and PE, with Fe–Mn binary oxide (FMBO) has resulted in the development of novel polymer-based adsorbents, PET-FMBO and PE-FMBO, that exhibit rapid kinetics in the removal of arsenic species. These nanocomposites have proven effective at removing both As(III) and As(V) across pH levels relevant for practical drinking water applications. Kinetic modeling revealed that the pseudo-second-order model provided the best fit, indicating that chemisorption is the dominant mechanism. The Freundlich isotherm model further supported multilayer adsorption on heterogeneous surfaces. A key advantage of these materials is their ability to oxidize As(III) to As(V) in situ, facilitating the simultaneous removal of both species without the need for a pre-oxidation step.

Despite a gradual decrease in adsorption efficiency after successive regeneration cycles, both PET-FMBO and PE-FMBO exhibited relatively high removal efficiencies for As(III) and As(V), even after multiple cycles, suggesting their potential for extended use in arsenic removal.

In comparison to powdered adsorbents, these granular FMBO nanocomposites offer inherent benefits by eliminating the need for complex post-treatment separation processes, making them more cost-effective for large-scale water treatment. However, further evaluation under continuous flow conditions using arsenic-contaminated groundwater is essential to fully assess their practical applicability and long-term performance.

An additional environmental advantage is the recyclability of PET and PE, which not only enhances the sustainability of these nanocomposites but also contributes to reducing the environmental impact of plastic waste. By aligning with circular economy principles, the use of recycled PET and PE as carriers for FMBO underscores the broader potential of this technology to promote both environmental sustainability and resource efficiency in water treatment solutions.

## Figures and Tables

**Table 1:** Textural properties of FMBO nanocomposites.

Fe–Mn Nanocomposites	BET (m^2^/g)	Mesopore Volume BJH (cm^3^/g)	Micropore Volume *t*-Test	Average Pore Size (nm)
PE	0.248	0	0	3.27
PE-FMBO	0.340	0	0	3.14
PET	0.325	0	0	7.21
PET-FMBO	0.389	0.001	0	7.78
FMBO	250	0.338	0	3.30

**Table 2:** EDS analysis of FMBO nanocomposites.

Fe–Mn Nanocomposites	Elements Weight (%)					
	C	O	Si	Ca	Mn	Fe
PE	100	-	-	-	-	-
PE-FMBO	78.0	5.80	-	1.05	2.02	5.78
PET	68.7	25.5	0.91	0.56	-	-
PET-FMBO	60.7	29.2	5.60	4.56	1.54	4.82
FMBO	6.48	27.6	-	-	17.6	50.0

**Table 3:** Parameters of Freundlich, Langmuir, Temkin, and Dubinin–Radushkevich adsorption isotherm models for As(III) and As(V) adsorption on PE-FMBO, PET-FMBO and FMBO.

Compound	pH	Material	Freundlich Model			Langmuir Model			Dubinin–Radushkevich Model				Temkin Model		
			R^2^	n	K_F_ (mg/g)/(mg/L)^n^	R^2^	q_max_ (mg/g)	K_L_ (L/mg)	R^2^	B	q_S_	E_a_	R^2^	b_T_ (J/mol)	A_T_ (L/g)
As(III)	6	FMBO	0.975	0.57	6.62	0.971	20.82	0.49	0.964	5.33 × 10^−4^	11.9	30.6	0.803	66.8	1263.1
		PE-FMBO	0.978	0.43	2.15	0.957	5.75	0.65	0.941	4.95 × 10^−4^	5.52	31.8	0.798	81.4	3803.5
		PET-FMBO	0.984	0.57	3.00	0.954	12.0	0.35	0.956	7.16 × 10^−4^	10.5	26.4	0.709	31.9	2014.4
	7	FMBO	0.988	0.42	5.81	0.953	11.0	2.08	0.965	2.88 × 10^−4^	11.6	41.7	0.826	132.8	1746.1
		PE-FMBO	0.985	0.42	2.03	0.977	5.29	0.72	0.72	4.60 × 10^−4^	5.06	33.0	0.712	180.4	4858.8
		PET-FMBO	0.955	0.55	2.31	0.925	8.74	0.38	0.910	5.89 × 10^−4^	7.449	29.1	0.598	131.6	3505.9
	8	FMBO	0.986	0.43	4.95	0.958	11.0	1.05	0.961	3.58 × 10^−4^	10.8	37.3	0.843	117.9	1927.3
		PE-FMBO	0.935	0.49	1.23	0.872	4.76	0.24	0.852	8.08 × 10^−4^	4.22	24.9	0.683	23.3	4531.8
		PET-FMBO	0.976	0.78	2.18	0.953	7.2	0.55	0.941	4.88 × 10^−4^	6.563	32.0	0.723	82.18	3318.1
As(V)	6	FMBO	0.999	0.79	11.1	0.996	46.2	0.33	0.990	5.08 × 10^−4^	28.6	31.4	0.789	30.18	746.7
		PE-FMBO	0.985	0.55	1.37	0.978	5.60	0.34	0.941	7.35 × 10^−4^	4.97	26.1	0.889	7.89	2737.8
		PET-FMBO	0.986	0.78	2.18	0.960	18.4	0.14	0.976	1.05 × 10^−3^	12.39	21.8	0.554	17.19	1931.6
	7	FMBO	0.994	0.69	10.5	0.985	29.7	0.60	0.981	4.29 × 10^−4^	24.1	34.1	0.866	33.0	761.6
		PE-FMBO	0.967	0.48	1.67	0.952	5.37	0.51	0.968	6.14 × 10^−4^	5.18	28.5	0.920	6.48	2315.7
		PET-FMBO	0.983	0.58	3.75	0.969	13.3	0.47	0.976	5.93 × 10^−4^	12.0	29.0	0.843	11.8	1223.7
	8	FMBO	0.972	0.63	7.87	0.945	22.4	0.66	0.954	5.05 × 10^−4^	21.4	31.5	0.730	22.4	865.0
		PE-FMBO	0.981	0.37	2.22	0.963	5.20	0.81	0.960	4.44 × 10^−4^	5.09	33.6	0.862	44.2	3512.7
		PET-FMBO	0.962	0.32	4.43	0.958	7.63	3.33	0.963	2.33 × 10^−4^	8.08	46.3	0.855	207.7	2567.1

**Table 5:** The concentration of manganese after adsorption of As(III) and As(V) on PE-FMBO, PET-FMBO, and FMBO.

	As(III)			As(V)		
Adsorbents	pH 6	pH 7	pH 8	pH 6	pH 7	pH 8
PE-FMBO	0.587	0.432	0.405	0.210	0.121	0.305
PET-FMBO	0.670	0.424	0.234	0.211	0.181	0.112
FMBO	0.521	0.423	0.321	0.364	0.212	0.232

## Data Availability

The data presented in this study are available on request from the corresponding author.

## Supplementary Materials

The following supporting information can be downloaded at https://www.mdpi.com/article/10.3390/ma17205089/s1, Table S1: Mathematical models used for modelling data obtained in kinetic and isotherm adsorption experiments; Table S2: Parameters of Lagergren pseudo-first order, pseudo-second order and Elovich model for adsorption of As(III) and As(V) on FMBO, PE-FMBO and PET-FMBO at pH 6, pH 7 and pH 8; Figure S1: FMBO nanocomposites (a) PE (b) PE-FMBO (c) PET (d) PET-FMBO (e) FMBO; Figure S2: Pore size distribution of of unmodified polymer and FMBO nanocomposite (a) PE (b) PE-FMBO; (c) PET (d) PET-FMBO and (e) FMBO calculated using BJH method; Figure S3: DFT pore size distribution analysis of the (a) PE (b) PE-FMBO; (c) PET (d) PET-FMBO and (e) FMBO; Figure S4: Point of zero charge of unmodified polymer and FMBO nanocomposite (a) PE and PE-FMBO (b) PET and PET-FMBO; Figure S5: Adsorption of As(III) and As(V) on PET-FMBO and PE-FMBO at different pH (m = 0.5 mg, V = 20 mL (0.1 M NaNO_3_), C_0_ As(III)/As(V) = 0.2 mg/ L, pH = 6—8, contact time 24 h). References [66,67,68,69] are cited in the supplementary materials.
