# Supplementary material for: Adsorption Performance of Fe–Mn Polymer Nanocomposites for Arsenic Removal: Insights from Kinetic and Isotherm Models
Source: Materials (Basel). 2024 Oct 18;17(20):5089. doi: 10.3390/ma17205089 (PMC11509112; doi:10.3390/ma17205089)
Supplement: Supplementary file 1 [file materials-17-05089-s001.zip › materials-3248314-supplementary.pdf]

Supplementary materials

Adsorption Performance of Fe-Mn Polymer Nanocomposites for Arsenic Removal: Insights from Kinetic and Isotherm Models

Jasmina Nikić[1], Malcolm Watson[1*], Jovana Jokić Govedarica[1], Maja Vujić[1], Jovana Pešić[1], Srđan Rončević[1] and Jasmina Agbaba[1]

[1*]University of Novi Sad, Faculty of Sciences, Department of Chemistry, Biochemistry and Environmental Protection, Trg Dositeja Obradovića 3, 21000 Novi Sad

*Corresponding author: malcolm.watson@dh.uns.ac.rs

Table S1. Mathematical models used for modelling data obtained in kinetic and isotherm adsorption experiments

Model	Equation	Linear form	Reference
**Pseudo-first-order**	$\frac{dq_t}{dt} = k_1(q_e - q_t)$	$\log(q_e - q_t) = \log q_e - \frac{k_1}{2.303}t$	Wang and Guo (2020)
**Pseudo-second-order**	$\frac{dq_t}{dt} = k_2(q_e - q_t)^2$	$\frac{t}{q_t} = \frac{1}{k_2 q_e^2} + \frac{1}{q_e}t$	
**Elovich**	$\frac{dq_t}{dt} = \alpha\ exp(-\beta q_t)$	$q_t = \frac{1}{\beta}\ln(\alpha\beta) + \frac{1}{\beta}\ln t$	Nanganoa et al. (2019)
**Freundlich**	$q_e = K_F C_e^{n_F}$	$\log q_e = \log K_F + n_F \log C_e$	Worch, 2012
**Langmuir**	$q_e = \frac{K_L C_L}{1 + \alpha_L C_e}$	$\frac{C_e}{q_e} = \frac{\alpha_L}{K_L}C_e + \frac{1}{K_L}$	Foo and Hameed (2010)
**Temkin**	$q_e = \frac{RT}{b_T}\ln(A_T C_e)$	$q_e = \frac{RT}{b_T}\ln A_T + \frac{RT}{b_T}\ln C_e$	
**Dubinin-Redushkevich**	$q_e = q_s\ exp(-B\varepsilon^2)$	$\ln q_e = \ln q_s - B\varepsilon^2$	

Table S2. Parameters of Lagergren pseudo-first order, pseudo-second order and Elovich model for adsorption of As(III) and As(V) on FMBO, PE-FMBO and PET-FMBO at pH 6, pH 7 and pH 8

Compound	pH	Material	Pseudo-first order			Pseudo-second order					Elovich		
			[a]k_1 (h^{-1})	[b]q_e (mg/g)	[c]R^2	[d]k_2 (g/mg h)	[e]h (mg/g h)	q_e (theoretical) (mg/g)	q_e (experimental) (mg/g)	R^2	[f]α (mg/gh)	[g]β (mg/g)	R^2
As(III)	6	FMBO	9.07	0.007	0.997	5729.3	229445.9	0.007	0.007	0.998	1.06E+04	1.15E+27	0.993
		PET-FMBO	5.43	0.006	0.980	2178.4	30608.0	0.006	0.006	0.984	4.72E+03	2.42E+08	0.951
		PE-FMBO	1.40	0.007	0.951	284.5	580.9	0.007	0.007	0.963	1.02E+03	1.02E-01	0.878
	7	FMBO	11.2	0.006	0.996	9092.2	524115.8	0.006	0.006	0.997	7.75E+26	1.16E+04	0.997
		PET-FMBO	4.47	0.005	0.993	1562.5	14404.3	0.006	0.006	0.997	1.91E+03	3.06E+03	0.957
		PE-FMBO	1.14	0.006	0.922	234.6	356.6	0.006	0.006	0.936	3.66E-02	9.76E+02	0.839
	8	FMBO	8.86	0.006	0.994	4825.1	145045.5	0.006	0.006	0.998	1.48E+14	7.03E+03	0.996
		PET-FMBO	7.82	0.005	0.915	6041.0	201080.8	0.006	0.005	0.979	2.17E+26	1.32E+04	0.970
		PE-FMBO	1.82	0.006	0.920	402.4	1079.9	0.007	0.006	0.932	2.25E-01	1.24E+03	0.810
As(V)	6	FMBO	10.8	0.007	0.931	6735.3	326622.7	0.007	0.007	0.999	1.16E+28	1.05E+04	0.997
		PET-FMBO	7.27	0.007	0.998	3425.2	79777.6	0.007	0.007	0.997	2.50E+14	6.55E+03	0.985
		PE-FMBO	2.00	0.007	0.985	400.9	1205.6	0.008	0.007	0.964	4.03E-01	1.17E+03	0.864
	7	FMBO	8.02	0.006	0.998	4477.3	128296.4	0.006	0.006	0.998	1.88E+17	8.07E+03	0.990
		PET-FMBO	8.31	0.007	0.991	3808.3	95720.8	0.007	0.007	0.998	1.39E+11	5.54E+03	0.997
		PE-FMBO	2.08	0.005	0.961	537.7	16220.9	0.056	0.006	0.989	2.69E-01	1.53E+03	0.949
	8	FMBO	5.48	0.006	0.948	2073.1	28365.1	0.007	0.006	0.995	8.93E+06	4.07E+03	0.970
		PET-FMBO	3.54	0.006	0.986	1054.7	7008.1	0.006	0.006	0.998	6.35E+01	2.29E+03	0.957
		PE-FMBO	1.39	0.007	0.956	290.0	597.1	0.007	0.007	0.987	1.01E-01	1.04E+03	0.905

[a]k_1 - rate constant of first-order sorption; [b]q_e - adsorption capacity; [c]R^2 - correlation coefficient; [d]k_2 – rate constant of second-order sorption; [e]h - initial adsorption rate; [f]α - initial chemisorption rate; [g]β – desorption constant

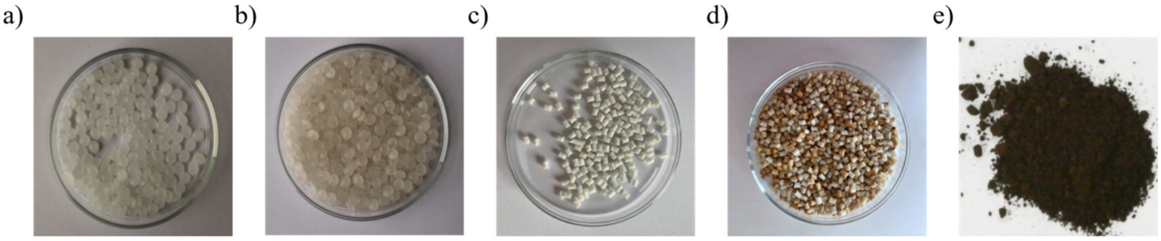

Figure S1. FMBO nanocomposites a) PE b) PE-FMBO c) PET d) PET-FMBO e) FMBO

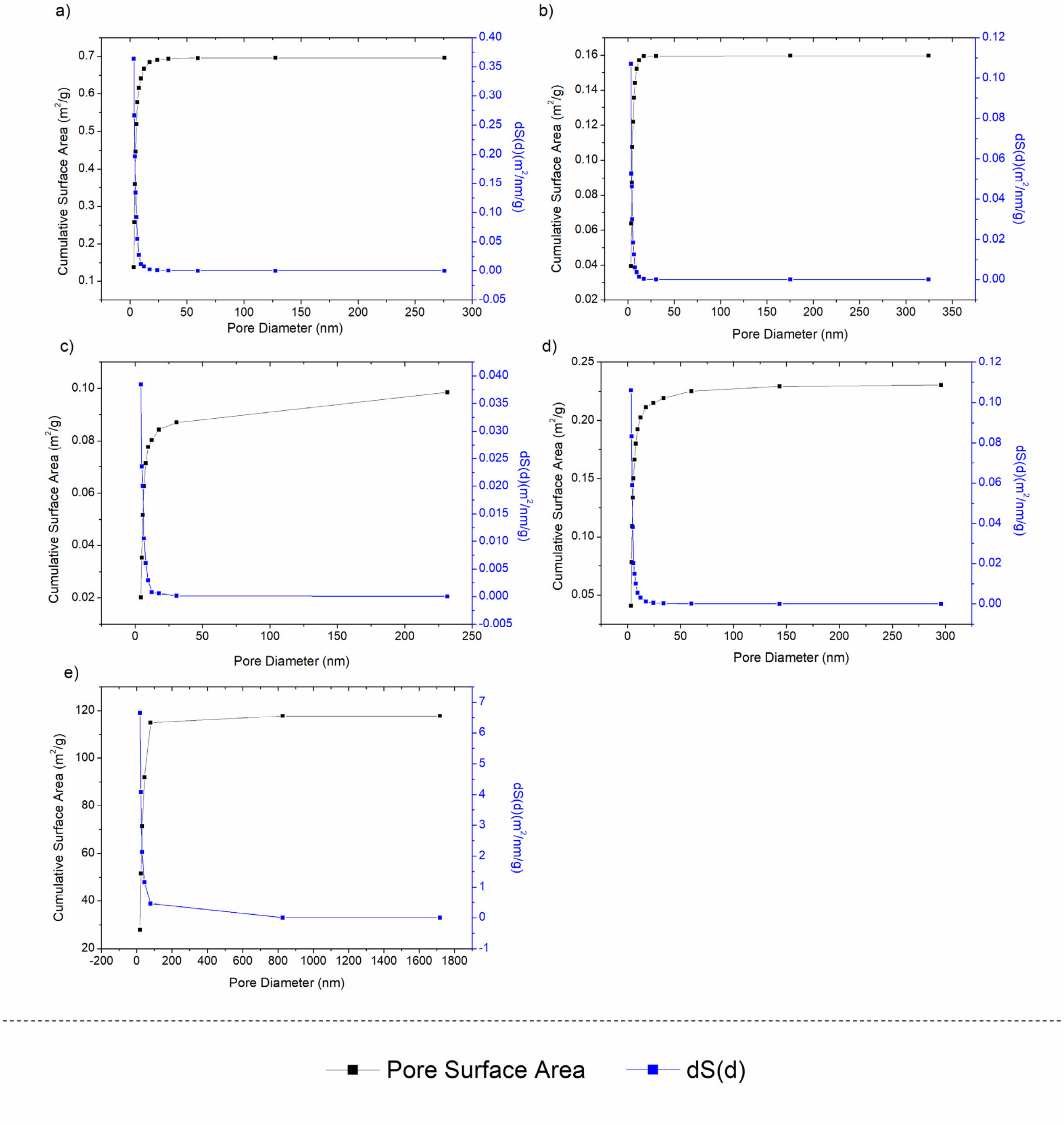

Figure S2. Pore size distribution of of unmodified polymer and FMBO nanocomposite a) PE b) PE-FMBO; c) PET d) PET-FMBO and e) FMBO calculated using BJH method

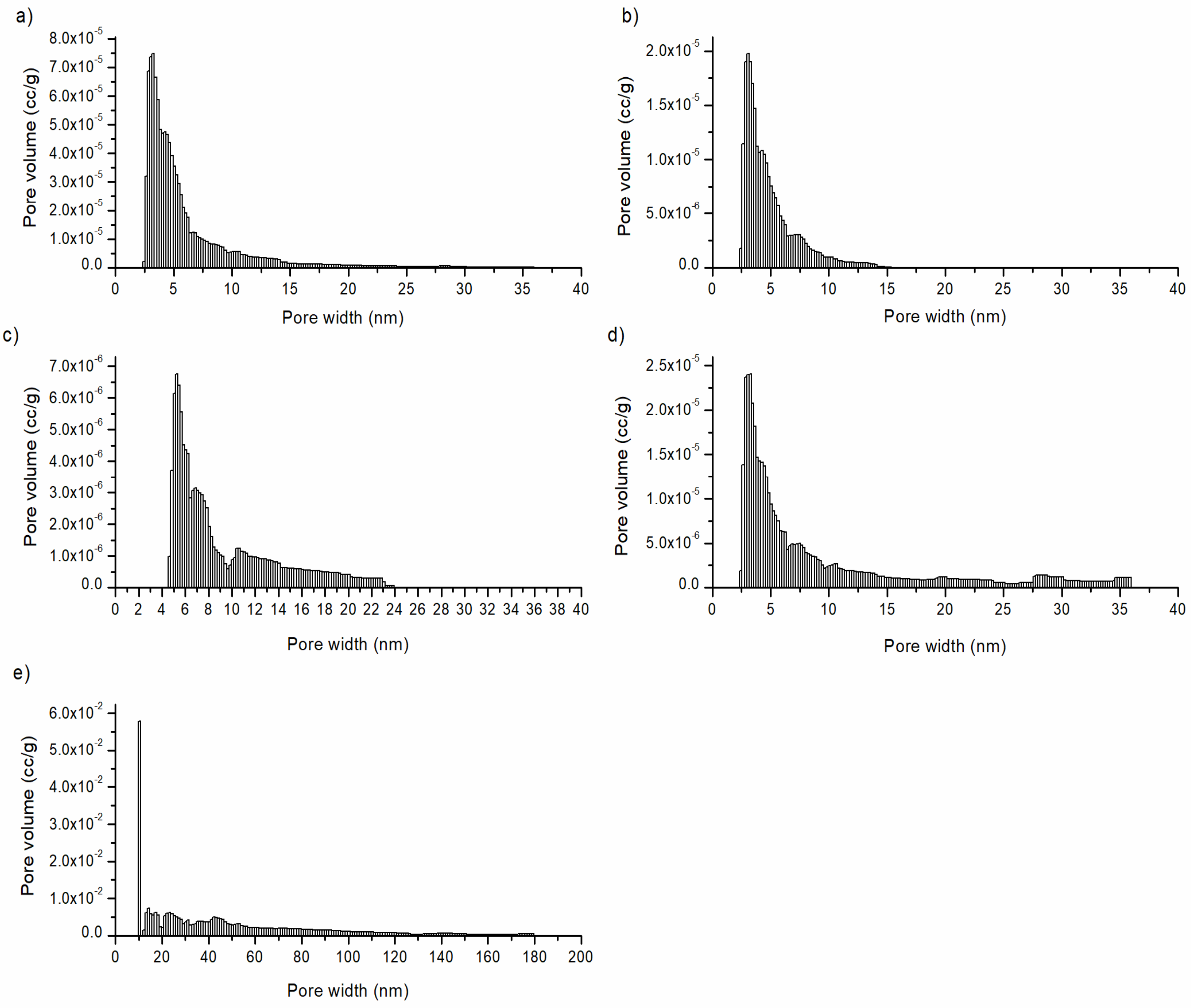

Figure S3. DFT pore size distribution analysis of the a) PE b) PE-FMBO; c) PET d) PET-FMBO and e) FMBO

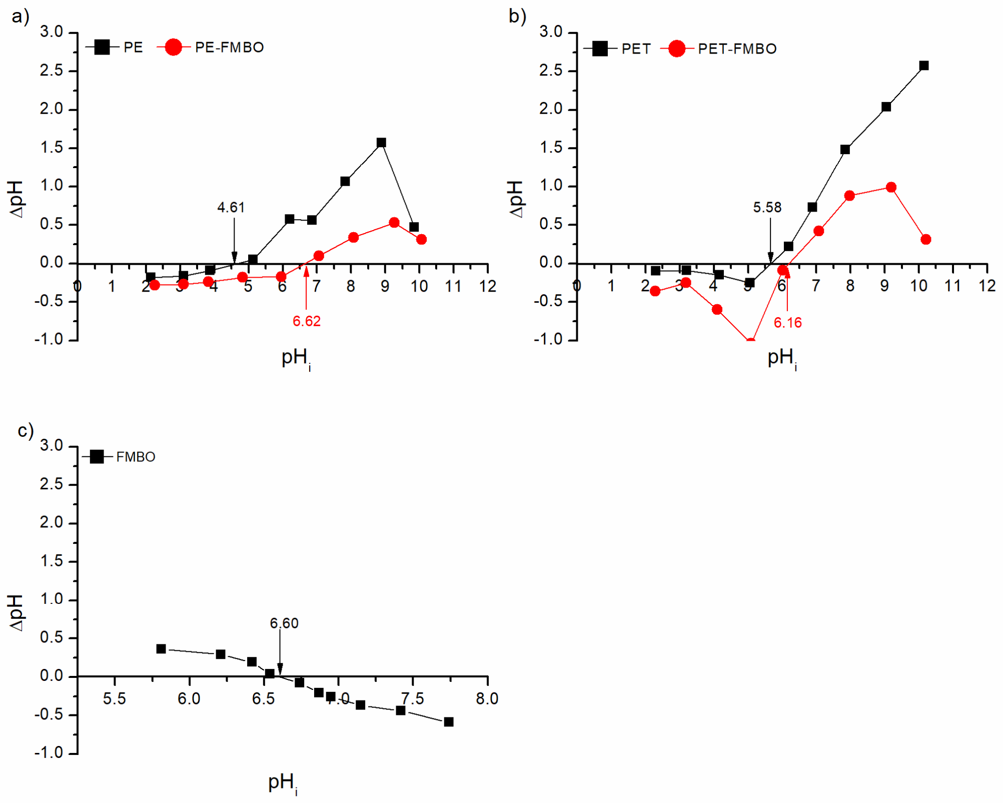

Figure S4. Point of zero charge of unmodified polymer and FMBO nanocomposite a) PE and PE-FMBO b) PET and PET-FMBO

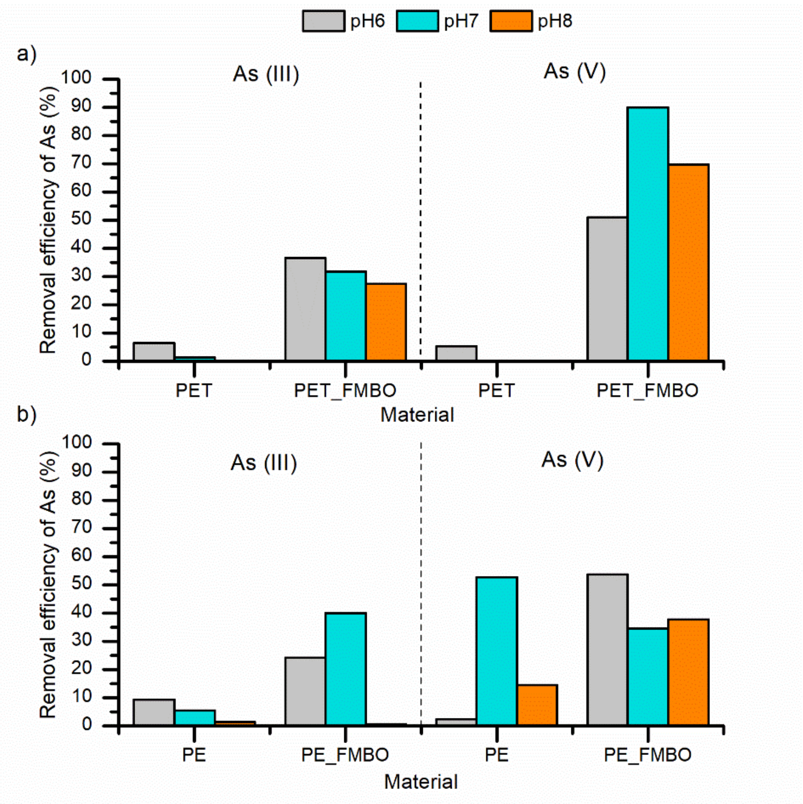

Figure S5. Adsorption of As(III) and As(V) on PET-FMBO and PE-FMBO at different pH (m = 0.5 mg, V = 20 mL (0.1 M $NaNO_3$), C_0 As(III)/As(V) = 0,2 mg/ L, pH = 6 - 8, contact time 24 h)

References

Nanganoa, L. T., Merlain, G. T., Ndi, J. N., Ketcha, J. M., 2019 Removal of ammonium ions from aqueous solution using hydroxy-sodalite zeolite, Asian J. Green Chem., 3(2), pp. 169-186. doi: 10.22034/ajgc.2018.140227.1083

Wang, j, Guo, X., 2020 Adsorption kinetic models: Physical meanings, applications, and solving methods. J. Hazard. Mater., 390, 122156. https://doi.org/10.1016/j.jhazmat.2020.122156.

Worch, E., 2012 Adsorption Technology in Water Treatment: Fundamentals, Processes, and Modeling. Berlin, Boston: De Gruyter. https://doi.org/10.1515/9783110240238

Foo, K.Y., Hameed, B.H., 2010 Insights into the modeling of adsorption isotherm systems. Chem. Eng. J. 156, 2–10. https://doi.org/10.1016/j.cej.2009.09.013
